# A Multimedia Support Skills Intervention for Female Partners of Male Smokeless Tobacco Users: Use and Perceived Acceptability

**DOI:** 10.2196/formative.9948

**Published:** 2018-05-28

**Authors:** Laura Akers, Judy A Andrews, Judith S Gordon

**Affiliations:** ^1^ Oregon Research Institute Eugene, OR United States; ^2^ College of Nursing University of Arizona Tucson, AZ United States

**Keywords:** tobacco cessation, social support, multimedia, website design, website development, website use assessment, usability testing

## Abstract

**Background:**

UCare is a new multimedia (website+booklet) intervention for women who want their male partner to quit their use of smokeless tobacco. The intervention is based on research showing that perceived partner responsiveness to social support is highest when the supporter conveys respect, understanding, and caring in their actions. The website included both didactic and interactive features, with optional video components, and special activities to help women develop empathy for nicotine addiction. The booklet reinforced the website content, encouraged women to use the website, and served both as a physical reminder of the intervention and a convenient way to share the information with her partner.

**Objective:**

The objective of this study was to describe the utilization and acceptability of a multimedia intervention among women seeking to support their partner in quitting smokeless tobacco. Lessons learned with respect to design considerations for online interventions are also summarized.

**Methods:**

We present the evaluation of the intervention components’ use and usefulness in a randomized trial.

**Results:**

In the randomized clinical trial, more than 250,000 visits were made to the website in a 2-year period, with the vast majority from mobile devices. Of the 552 women randomized to receive the intervention, 96.9% (535/552) visited the website at least once, and 30.8% (170/552) completed the core website component, “The Basics.” About half of the women (287/552) used the interactive “Take Notes” feature, and 37% (204/552) used the checklists. Few women used the post-Basics features. At 6 weeks, 40.7% (116/285) reported reading the printed and mailed booklet. Website and booklet use were uncorrelated. User ratings for the website and booklet were positive overall.

**Conclusions:**

Intervention website designers should consider that many users will access the program only once or twice, and many will not complete it. It is also important to distinguish between core and supplemental features and to consider whether the primary purpose is training or support. Furthermore, printed materials still have value.

**Trial Registration:**

ClinicalTrials.gov NCT01885221; https://clinicaltrials.gov/ct2/show/NCT01885221 (Archived by WebCite at http://www.webcitation.org/6zdIgGGtx)

## Introduction

### Background

Approximately 8.2 million Americans regularly use smokeless tobacco (ST), which increases their risk of head and neck cancers [1], as well as cardiovascular and cerebrovascular mortality [2,3]. The vast majority of ST users are males; 4.8% of males and 0.3% of females use ST “every day” or “some days” [4]. ST use is highly addictive [1], and few resources exist to help users quit. As a novel approach toward facilitating ST users’ cessation, we developed an intervention targeting male ST users’ wives and female domestic partners to help motivate their partners to quit and support them during quit attempts. We found in our previous research [5-9] that that women were enthusiastic about the prospect of helping their partners quit their use of ST.

With support from the National Institute on Drug Abuse (NIDA, grant 1R01DA033422), we developed an intervention for women based on a framework of perceived partner responsiveness [10], indicating that support is best received when it conveys respect, understanding, and caring. We named the intervention with an acronym for these themes: UCare. We then conducted a randomized clinical trial (RCT), enrolling 1145 women in 15 months using Facebook advertising [11], randomizing 552 women to the intervention condition and the rest to Delayed Treatment Control. We administered online assessments at baseline and at 6 weeks and 7.5 months postenrollment.

In this paper, we present data on the use and perceived acceptability of the intervention features. We then discuss the lessons learned from this process and their applicability to future eHealth intervention design.

#### Intervention Content: UCare Website and Booklet

The UCare intervention is a multimedia program, featuring both an interactive, mobile-optimized website and a printed booklet. The multimedia approach has several benefits: 1) the intervention fits a variety of preferences for accessing information (audio, text, etc); 2) the mobile-optimized website is easily accessible from anywhere at any time; 3) the booklet provides the women with material she can easily show her partner to reassure him that the program is not focused on trying to “make him” quit; and 4) the public health approach is cost-effective and sustainable over time. The website also offers the opportunity to track intervention use, whereas data on use of the booklet relies on the users’ self-report.

#### Intervention Structure

**Figure 1:**
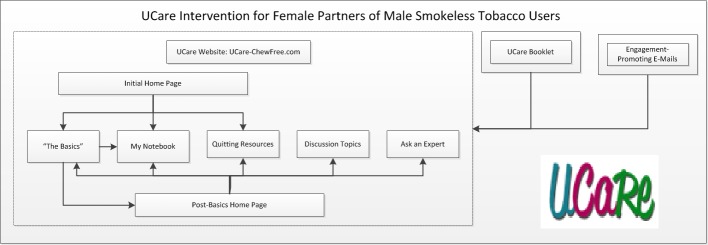
UCare intervention schematic.

The UCare website provided a concise instructional program (“The Basics”), with options for personal tailoring and printable lists of decisions and chosen activities (“My Notebook”), supplemented with ST cessation resources (“Quitting Resources”), social support forums, and email reminders to remain engaged with the program. Figure 1 provides an overview of the intervention.

### Website Content

#### The Basics

**Figure 2:**
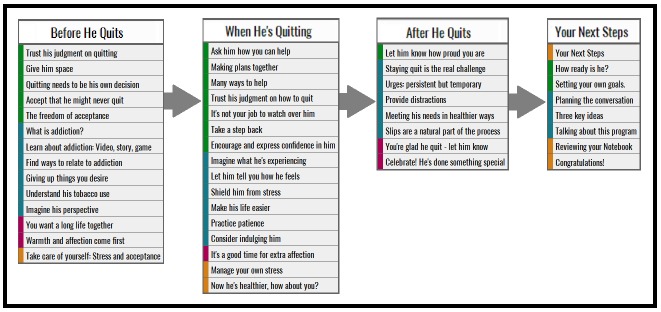
Structure of the UCare “Basics.”

The core of the intervention was a four-module linear website component that we called “The Basics.” We designed the first three modules to teach users about the three main stages of quitting (planning, quitting, and maintenance), and the fourth module helped the user create a plan for how to approach her partner about quitting. Within each section, we presented information sequentially, starting first with how to convey respect at that stage, followed by how to convey understanding, followed by how to convey caring (see Figure 2). Progress through “The Basics” was linear (“tunnel” architecture, such that each page could be accessed only after completing the previous page), but once a page had been visited, it could be accessed directly from the menu thereafter (random access). The website software system tracked each user’s progress through “The Basics” so that she would automatically return to the furthest page she’d reached when she next logged in. Each Basics section was color-coded, with each color indicating the respective responsiveness theme (respect, care, and understanding), to allow for easy discrimination between sections. A fourth theme was self-care, including stress reduction for the caregiver, as stress management and patience have been found to be key to providing support to others in many contexts [12-14].

**Figure 3:**
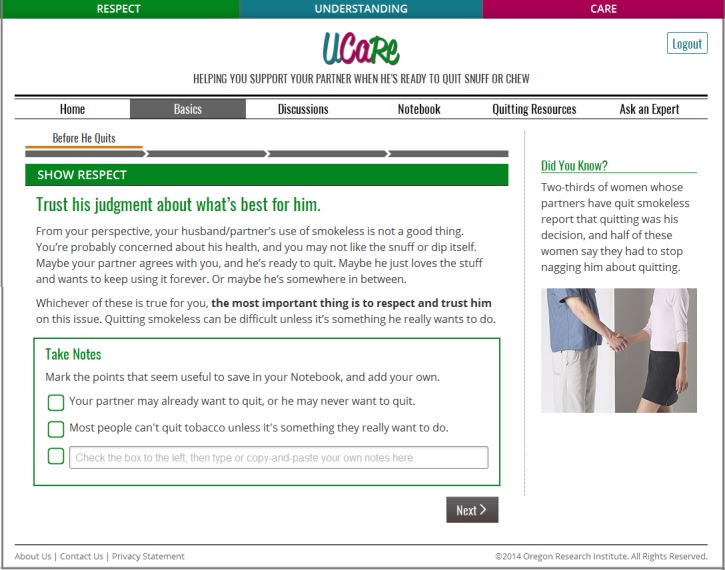
UCare Basics sample text page.

Most of the 46 pages in “The Basics” were either text plus a stock image (31 pages) or testimonials (10 pages); see Figure 3. Each testimonial page included 3 quotes from women, each associated with a photograph of a woman, with the text crafted from formative interviews (see Multimedia Appendix 1, Figure A-1). Users could click an audio link to hear the quote in the woman’s voice. Video clips were not used because they would require more bandwidth and be less compatible with mobile phone use of the program.

#### “Learn About Addiction” Features

Previous formative work [15] suggested that women often experience difficulty in understanding addiction. We used three approaches to teach women what addiction is and what it is like: a didactic approach (presenting scientific information), a narrative immersion approach, and an experiential approach. One Basics page provided links to three special features using these approaches. A “Nicotine in the Brain” video featured a 41-second animation, with accompanying text matching the animation audio, as a didactic approach to explaining addiction (Multimedia Appendix 1, Figure A-2). The video was inspired by a short video developed by the Mayo Clinic. “Megan’s Morning” (Multimedia Appendix 1, Figure A-3) was a 1200-word fictional story in text and audio formats about the experiences of a woman trying to quit smoking (third grade reading level). This story, written for the website by award-winning author Nina Kiriki Hoffman, provided a narrative immersion approach to help the reader or listener empathize with the quitting process. To provide an experiential approach to conveying the challenge of addiction, “The CONCENTRATE Game” (Multimedia Appendix 1, Figure A-4) illustrated the disruptions to concentration that nicotine cravings can cause and the effect of tobacco on alleviating the cravings while reinforcing addiction. Playing a game involving perspective-taking has been shown to build empathy in other contexts [16]. The project team extensively tested the special features for their functionality across a wide variety of platforms.

#### My Notebook

Retention and use of intervention information has been shown to be greatest when the user can personalize their experience and create their own plan for using the information [17,18]. Furthermore, meta-analyses have found that goal-setting and action planning via internet interventions are associated with behavior change [19]. The “My Notebook” feature, accessible from the Main Menu, was an interactive tool allowing the user to create and print her own supporter plan, organized into 2 pages, “Things to Remember” and “Things to Do.” “Things to Remember” was populated by choices made throughout “The Basics,” as most pages (both text and testimonials) included a “Take Notes” feature allowing her to save key points from the program (her choice of presupplied notes and her own text entries). “Things to Do” was created from 4 interactive checklists in “The Basics” (see Multimedia Appendix 1, Figures A-5 and A-6), with ideas for managing stress, setting goals the participant could achieve without the ST user’s participation, and working with her partner to plan how she could best support him if he decided to try to quit (see Multimedia Appendix 1, Figure A-2)

#### Quitting Resources

We provided a main menu link to 16 pages of quitting information for women who wanted more information about addiction and the quitting process. Pages in this section describe smokeless tobacco and its contents, explain how nicotine addiction works, describe the quitting process, present information on how to access quitlines and the ChewFree.com ST cessation program, and describe quitting aids for ST users.

#### Discussion Topics

Based on our experiences with ST users, we anticipated that long-term engagement with the intervention would be desirable and helpful for this population, and we wanted to give the women the opportunity to develop a mutually supportive community. This type of asynchronous mediated peer-to-peer communication has been associated with relatively more effective eHealth interventions [20]. We also wanted to be able to add new content to the website if users identified needs we had not anticipated. For these reasons, we created a section of Discussion Topics, which included text explaining a topic (eg, how to quit tobacco with your partner, how to talk to your children about their father’s quit attempt, how to broach the topic of quitting with your partner), and then a threaded comments section (see Multimedia Appendix 1, Figure A-7). New Topics were added with an email announcement and a direct link to that page. A “Suggest a Topic” button allowed users to propose ideas for new “Discussion Topics.”

#### “Ask an Expert” Forum

This feature provided users with the opportunity to ask questions of our staff, either about providing support or quitting ST.

#### Marketing Page and Enrollment Process

**Figure 4:**
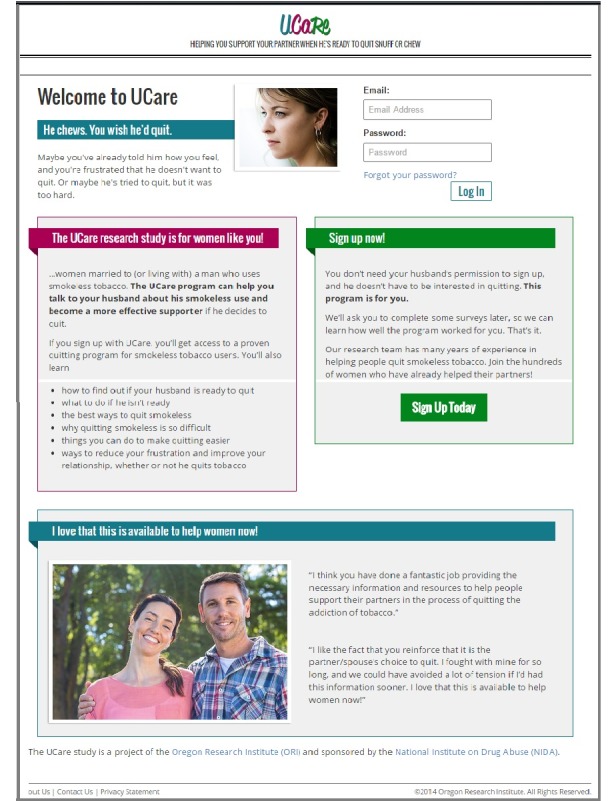
UCare home and marketing page.

We created the study homepage with two functions: marketing information explaining the study to new visitors and a login option for registered users (see Figure 4). Women who registered with the study and were randomized to intervention began their use of the UCare website with an animated tour of the main menu and website functions which invited them to begin “The Basics.” They received up to 8 reminders to finish “The Basics” and notices of new “Discussion Topics.”

### Booklet Content

We adapted our existing supporter booklet, developed in a previous pilot study (National Cancer Institute; NCI 1R21CA131461), to better match the themes, content, and graphics of the website. Three goals for the booklet were to summarize the essence of the intervention content (focusing on realistic goal-setting depending on her partner’s readiness to quit and providing quick lists of support do’s and don’ts); to provide a means for the women to show their partner what the UCare program is about and to allay any concern on his part that the intervention might be coercive; and to encourage the women to use the website. The booklet gives an overview of the three responsiveness themes and numerous examples of how to use them in conversations.

### Formative Testing and Refinement

Throughout the formative testing, participants were primarily drawn from a pool of female partners of ST users who had participated in our previous research. For the overall pool, demographics were similar to the target population: the women were 94.5% (493/522) white, with 85.3% (436/522) attending some college or greater and 34.4% (180/522) having earned at least a bachelor’s degree, and with a mean age of 43.9 years (SD 7.4 years). First, 49 women reviewed initial webpage designs, then 17 women provided feedback on the “Learn About Addiction” features; based on their feedback we added an audio track for the story.

We then conducted in-person usability testing with a new sample of 12 women from the local area who had been in a long-term relationship with a male ST user. Each individual testing session involved the user sitting with a research assistant and using each of the website features, while “thinking out loud” as they made their choices. We made minor text changes and a few tweaks to the graphics based on their feedback.

Finally, 70 women signed up to beta test the website, and within 2 weeks, 46 of the women completed the 2- to 3-hour beta testing process. This involved going through the UCare enrollment process and creating an account, reading all of “The Basics” and any other website content of interest, making at least 1 post each on a “Discussion Topic” and in the “Ask an Expert” forum (seeding these features in an attempt to create a norm for their use among RCT participants), reading the printed booklet (which we mailed to them), and completing an online follow-up survey giving their reactions to each of the intervention components.

To assess the beta testers’ experiences with the website and booklet, we administered a user satisfaction measure with items adapted from Brooke’s [21] widely used and validated System Usability Scale [22]. Items in the measure were generally in 5-point Likert-scale format. The measure asked users to rate the website overall (eg, ease of use, helpfulness, desire to keep using, willingness to recommend to others), specific website components, and the acceptability and ease of use of the booklet. It also included open-ended questions about potential improvements to the website and booklet, especially to streamline the enrollment process and to include the website URL in the booklet.

### Randomized Trial: Assessment of Program Use and Acceptability

Enrollment for the RCT took place between July 2015 and December 2016. Inclusion criteria for the RCT were (a) being the wife or female domestic partner (living together) of a male currently using ST; (b) being interested in having him quit; (c) willing to provide a phone number, mailing address, and email address; (d) and willing to give informed consent. Additionally, the woman and ST user were (e) both US or Canadian residents aged 18 years or older, (f) both able to read English, and (g) both able to access a computer.

The women’s mean age was 43.2 years (SD 9.5) and the sample was 95.3% white (1081/1145), 96.3% non-Hispanic (1100/1142), and 87.8% (1001/1140) having completed some college; 25.6% (293/1145) of the women had ever used tobacco; the mean length of the relationship between the participant and the ST user (her husband or domestic partner) was 15.6 years (SD 10.3). The female participants were racially and ethnically similar to ST users but likely better educated (Cheng and colleagues [23] report 53.1% of ST users with some college education).

During the RCT, we unobtrusively tracked participants’ use of the website, recording all page hits and all choices made within the website, using standard methods that created a time-stamped archive of each user’s activities. An “admin site” with a user-friendly interface allowed the research staff to find contact information, assessment tracking information, and site use details for each participant. Information such as how many pages the user had visited was available both through the admin site and in a downloadable Excel spreadsheet.

Participants were asked to complete follow-up assessments at 6 weeks and 7.5 months postenrollment. We assessed perceived acceptability of the program components (and self-reported use of the booklet) at 6-week follow-up, using the measure that had been used with the beta testers. Women randomized to Delayed Treatment received access to the intervention after completing the 7.5-month follow-up.

## Results

### Website Use

Upon enrolling, women randomized to the intervention were immediately given access to the website, and almost all of the women eventually visited the website at least once (96.9%). Over the course of the 2-year RCT, more than 250,000 visits were made to the website, and 74.9% (190,968/254,915) of visits were from smartphones, 9.7% (24,728/254,915) from phone-tablets, or “phablets,” 8.5% (21,739/254,915) from tablets, and only 1.9% from desktops (4895/254,915), with 4.8% (112,297/254,915) from unidentifiable devices.

Table 1 shows the rate of intervention component use. Basics completion patterns were associated with the weekly reminder email—almost one-third of women (31.5%) who completed “The Basics” after their enrollment day did so on a day when they had received such an email, and the email with the “UCare final reminder” header (at 8 weeks after the last website visit) was especially effective at getting women to complete “The Basics.”

By the 6-week follow-up, 45.8% of the women used the “Take Notes” feature to populate “My Notebook” with “Things to Remember,” and 30.8% used the checklists within “The Basics” to choose “Things to Do.” These rates increased to 52.0% and 37.0% by the 7.5-month follow-up.

The three “Learn About Addiction” features each required the user’s choice to access them. The CONCENTRATE game was the most popular of these features; 24.3% of the women used it by 6-week follow-up. The game functions differently depending on whether the user clicks the “take a dip” feature or does not, and women were encouraged to try it both ways; 38.8% of those who tried it did so. By 6-week follow-up, 16.7% of the women watched the video, and 12.5% of the women accessed the “Megan’s Morning” story; for those accessing the story, 81% (111/137) chose to listen (the default), 12% (17/137) chose to read, and 7% (9/137) chose to listen while reading. Use of the three Learn About Addiction features was highly correlated (viewing the video and reading the story, r=.72; viewing the video and playing the game, r=.54, and reading the story and playing the game, r=.50).

**Table 1:** Use of UCare intervention components (N=552) and usability ratings (1-5).

Intervention component	Component usage, n (%)		*P* value^a^		Usability rating at 6-week follow-up, mean (SD)		Usability rating, n (%)	
	By 6-week follow-up	By 7.5-month follow-up						
UCare website (any use)	529 (95.8)	535 (96.9)		N/A^b^		3.79 (.89)		259 (46.9)
The Basics (any use)	346 (62.7)	387 (70.1)		<.001		3.98 (.78)		217 (39.3)
The Basics (completion; 46 pages)	105 (19.0)	170 (30.8)		<.001		4.28 (.61)		94 (17.0)
Notebook entries (Take Notes)	253 (45.8)	287 (52.0)		<.001		3.58 (.86)^c^		170 (30.8)
Notebook entries (Checklist Choices)	170 (30.8)	204 (37.0)	<.001		3.58 (.86)^c^		170 (30.8)	
"Nicotine in the Brain" video	92 (16.7)	98 (17.8)		.01		3.85 (.87)^c^		62 (11.2)
"Megan’s Morning" fictional story	64 (12.5)	68 (13.2)		.045		3.49 (1.12)^c^		49 (8.9)
CONCENTRATE Game	134 (24.3)	146 (26.4)		.001		3.46 (1.18)		97 (17.6)
Basics testimonial audio (default printed text)	60 (10.9)	69 (12.5)		.003		N/A		N/A
Quitting Resources	56 (10.1)	96 (17.4)		<.001		4.06 (.08)		49 (8.9)
Post-Basics Discussion Topics	36 (6.5)	80 (14.5)		<.001		3.87 (.92)		31 (5.6)
Post-Basics Ask an Expert	5 (0.9)	17 (3.1)		.001		4.20 (.84)		5 (0.9)
UCare printed booklet (self-report at 6-week follow-up)	116 (40.7	N/A		N/A		3.89 (.78)		252 (45.7)

^a^Pairwise *t* tests between 6 weeks vs 7.5 months.

^b^N/A: not applicable.

^c^Ratings for the three Learn About Addiction features are the mean of two items: how much did the feature help you understand addiction and how much did it help you understand how hard it is to quit.

**Table 2:** Co-use of website “Basics” and printed booklet by 6-week follow-up.

Basics use	Self-Reported booklet use				
	“None”	“Some” or “Most”	“All” or “More Than Once”	Unknown^a^	Total
None (0-1 pages)	10	37	33	160	240
Some (2-45 pages)	14	63	37	96	210
Basics (all 46 pages)	9	36	46	11	102
Total	33	136	116	267	552

^a^Combines participants randomized to the intervention who did not complete the 6-week follow-up assessment and those who skipped that item while completing that assessment.

Completing “The Basics” typically takes 20-40 minutes, and for many women, this was all they did with the website. Very few read all of the Quitting Resources pages. Although 60.2% (332/552) of women returned to the UCare website after completing “The Basics,” use of the post-Basics features was very low. Only 1 woman made a comment on the Discussion Topics, none suggested new topics, and none made posts to Ask an Expert.

In the 6-week follow-up survey, 40.7% (116/285) of respondents reported that they had read all of the UCare booklet at least once. Booklet use was not asked about in the 7.5-month survey. Table 2 compares self-reported use of the booklet with automatically tracked use of “The Basics.”

Chi-squares and paired *t* tests indicated that use of every website component was significantly greater by 7.5-month follow-up than by 6-week follow-up. This finding applied both to whether the component had been used (yes or no) and how many times it had been used (eg, pages of a section read, notebook entries made, times video viewed, times game played).

### Understanding Use of Intervention Features

For the purpose of understanding use of the various aspects of the intervention, we used factor analysis to reduce 12 website use variables to a more manageable number. Using principal components analysis with varimax rotation, we identified three factors. Variables which loaded >.34 on the relevant factor were considered indicators of that factor. The factors were: Interactive Engagement (overall website engagement and use of the interactive features; 6 items), Audio Preference (use of the optional audio features; 3 items), and Thoroughness (use of the Quitting Resources and post-Basics features; 3 items—see Table 3). We created scales through standardizing the variables that loaded on each factor and summing them.

**Table 3:** Factor loadings of website exposure variables by 6-week follow-up (principal components analysis). Factor loadings>.340 are indicated in italics.

Website exposure variable	Factor 1: Interactive Engagement	Factor 2: Audio Preference	Factor 3: Thoroughness
Number of Notebook entries	.*907*	.121	.121
Number of Checklist Choices	.*885*	.023	.084
Basics pages completed	.*829*	.170	.321
Total minutes engaged in website	.*663*	.*406*	.311
Total visits	.*519*	.220	.*507*
Times played CONCENTRATE Game	.*426*	.*348*	–.*382*
Times viewed brain video	.136	.*818*	.088
Times listened to/read “Megan’s Morning”	.141	.*804*	–.038
Number of testimonial audio feature uses	.048	.*536*	.006
Quitting Resources visited	.013	.270	.*728*
Discussion Topics read	.*379*	–.085	.*521*
Expert forum threads read	.137	–.095	.*387*

The scale measuring Interactive Engagement was moderately correlated with Audio Preference (r=.304, *P*<.001) and with Thoroughness (r=.372, *P*<.001). The correlation between Audio Preference and Thoroughness (r=.061) was not significant, and none of the website exposure scales was correlated with the use of the printed booklet.

We then predicted each of the three scales along with booklet use from baseline variables for the RCT participants. “Interactive Engagement” was more common for the women with younger partners (standardized β=–.142, *P*=.02). “Thoroughness” was more common for women who were white (standardized β=.132, *P*=.03) and for women whose partner was not White (standardized β=–.208, *P*=.001). “Audio Preference” and use of the printed booklet were not correlated with baseline variables.

### Program Perceived Acceptability

At 6 weeks, 297 (53.8%) of women randomized to the intervention completed a follow-up assessment that included measures of consumer satisfaction with intervention components. For the website components, the calculations of mean satisfaction ratings, shown in Table 1, were limited to those women whose use of the rated components was verified by website tracking. For the booklet, the mean satisfaction rating was limited to the women who had reported at least some use of the booklet by 6-week follow-up. All components were rated as “somewhat helpful” or better, and the website and booklet were rated as “helpful” overall.

## Discussion

### Principal Findings

The UCare intervention is a multimedia program for women who want their male partner to quit his use of smokeless tobacco. Both online and print components had value. Some women used both, and others used the medium they preferred. The program accommodated different personal preferences for text vs audio; it also accommodated bandwidth limitations by using text-plus-audio rather than video to present interview content. Website and booklet use were uncorrelated (but note that only a third of the women randomized to the intervention provided booklet usage data). Participants generally rated all of the intervention components favorably.

Our experiences in developing the program yielded several findings that may benefit others who are developing eHealth interventions. First, changing trends in how people access websites must be taken into account. More than 90% of visits to our website were made via mobile devices; optimizing websites for mobile device use is vital.

Second, it is important to take into account that many users will access the intervention only once or twice and will not complete the intervention as it is intended. On average, our participants spent only 16 minutes on the website within 6 weeks of enrollment, whereas our core program takes 20-40 minutes to complete. If it is likely or plausible that many users will engage with the intervention only once, the most important information and key takeaway message should be presented early in the program. Throughout our website, booklet, and follow-up emails, we emphasized the value of completing “The Basics,” but only some 30% (170/552) of participants randomized to intervention did so.

Further, many women were not ready to use the intervention when they signed up for the study, and many women continued to access the intervention well after the initial 6-week period. Use of all intervention components was significantly higher at 7.5-month follow-up than at 6-week follow-up. This finding is relevant for at least two reasons: prompts designed to increase engagement (eg, emails) should continue past the initial use period, and assessments of exposure-related outcomes should be timed accordingly.

It is also critical to distinguish the core program from “extra” features, and to maximize users’ exposure to the core message. Intervention designers should consider using “tunnel” architecture to force users through the core program in its intended order, but make the pages directly accessible after their initial viewing for ease of use. The core program can then be supplemented as needed. For example, although our “Learn About Addiction” features were embedded in the core part of the program, they were optional and many skipped them. Likewise, few used the “Quitting Resources,” but the information could be readily accessed later, and many returned to read them.

Because our program consisted of many different components and activities, we were unable to discern which parts of the intervention contributed to program efficacy. Future studies could address this problem by using the Multiphase Optimization Strategy (MOST) developed by Collins et al [24-26] to refine potential intervention components in a preliminary trial prior to inclusion in the final program. Members of our team have used this approach successfully in other studies [27,28]. Strecher et al used this approach in developing a Web-based smoking cessation program for smokers ready to quit at two health maintenance organizations (HMOs) [29], and McClure and colleagues used MOST to explore components for new websites at the same HMOs, for smokers at any stage of readiness [30-32]. These studies tested specific program components for preliminary efficacy with their intended population prior to conducting a randomized efficacy trial of the overall program.

At the outset, intervention designers should consider whether their primary purpose is to provide training or support. If the former, users should be expected to visit only once or twice, and a core training module may be all that is needed. However, for lifestyle changes, ongoing engagement with the intervention may be desirable to produce a dose-response relationship [33]. In this case, the intervention information may be supplemented by creating a community for peer support and an opportunity to ask questions of experts. These features were popular in our ChewFree.com cessation program for ST users [34], but the UCare participants didn’t find them helpful and generally ignored them. Not only were these features apparently unwanted by the users, but they also represented a potential ongoing cost to our staff in terms of monitoring, maintenance, and crafting “expert” responses. It may have been easier for these women to find the peer support they needed among their friends and on social media than it had been for the ChewFree.com users, or they may have felt that the information in “The Basics” was all they needed. For this population, we could have omitted the “Discussion Topics” and “Ask an Expert” features. Other options would have included incorporating the information from the “Discussion Topics” into “The Basics.” Conversely, if we believed that adding new information throughout the study was important, we could have allowed users to access the post-Basics content (and potential community) from the beginning. We recommend that others weigh these features carefully when designing their own interventions.

Finally, eHealth intervention designers should consider whether the need they are addressing is an urgent one, such that participants will immediately make use of the program, or whether participants are more likely to wait for a convenient time. When an intervention is delivered soon after enrollment (eg, in-person or through phone counseling for tobacco cessation), effects are expected to follow soon thereafter, and traditional short-term and long-term follow-up assessments should be able to capture behavior change and assess whether it is being sustained. For an intervention like UCare, however, many study participants were not ready to access or complete the intervention until after the 6-week assessment. It is important to keep reminding participants that the intervention is available (we sent up to 8 reminder emails plus the assessment prompts), and measure use and its mediated effects accordingly.

### Limitations

User feedback on the usability of intervention components was collected only at 6 weeks, as we anticipated that retrospective recall at 7.5 months would be inaccurate. However, many women first used the website features after the 6-week assessment. Furthermore, it should be noted that the sample was self-selected, with women choosing to use the components they were rating. Usage data for the booklet were necessarily self-reported. Further analyses are necessary to connect the use of the intervention components to clinically meaningful outcomes.

### Conclusions

Today’s technology offers many opportunities for eHealth intervention. Researchers designing such interventions should take into account the behavior of users to ensure that key content is delivered most effectively.

## Appendix

Multimedia Appendix 1 UCare Development Appendix of Figures.

## Abbreviations

HMO: health maintenance organization
MOST: multiphase optimization strategy
NCI: National Cancer Institute
NIDA: National Institute on Drug Abuse
RCT: randomized clinical trial
ST: smokeless tobacco
