# Supplementary material for: A Multimedia Support Skills Intervention for Female Partners of Male Smokeless Tobacco Users: Use and Perceived Acceptability
Source: JMIR Form Res. 2018 May 28;2(1):e10. doi: 10.2196/formative.9948 (PMC6294132; doi:10.2196/formative.9948)

Appendix of Figures

UCare Development Paper, Laura Akers, et al.

Figure A-1. UCare Basics: Sample Testimonial Page

Each “testimonial” page featured text drawn from formative interviews, with optional audio files for those preferring to listen.

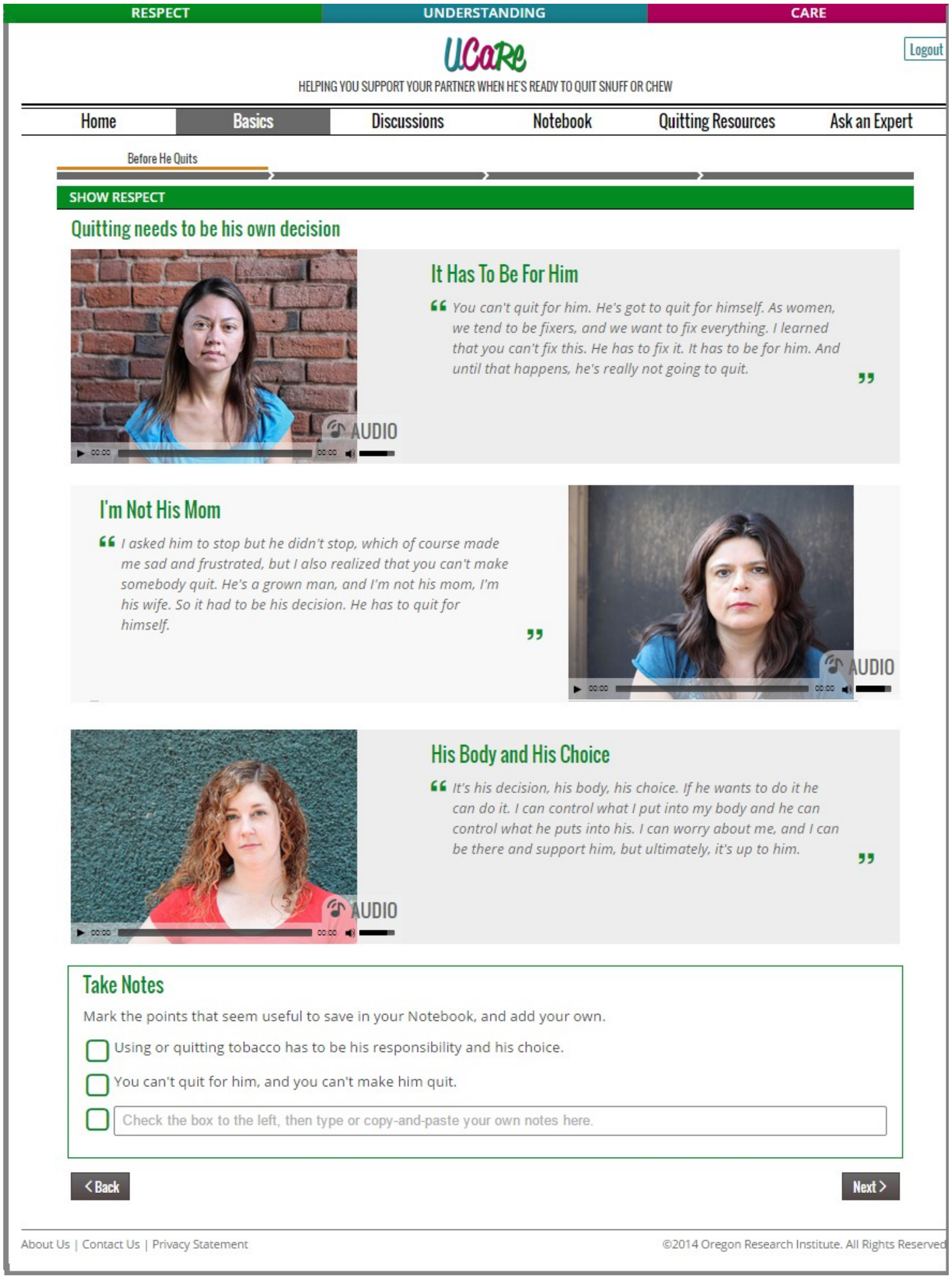

Figure A-2. “Nicotine in the Brain” Video

This animated video is accompanied by both text and an audio voiceover.

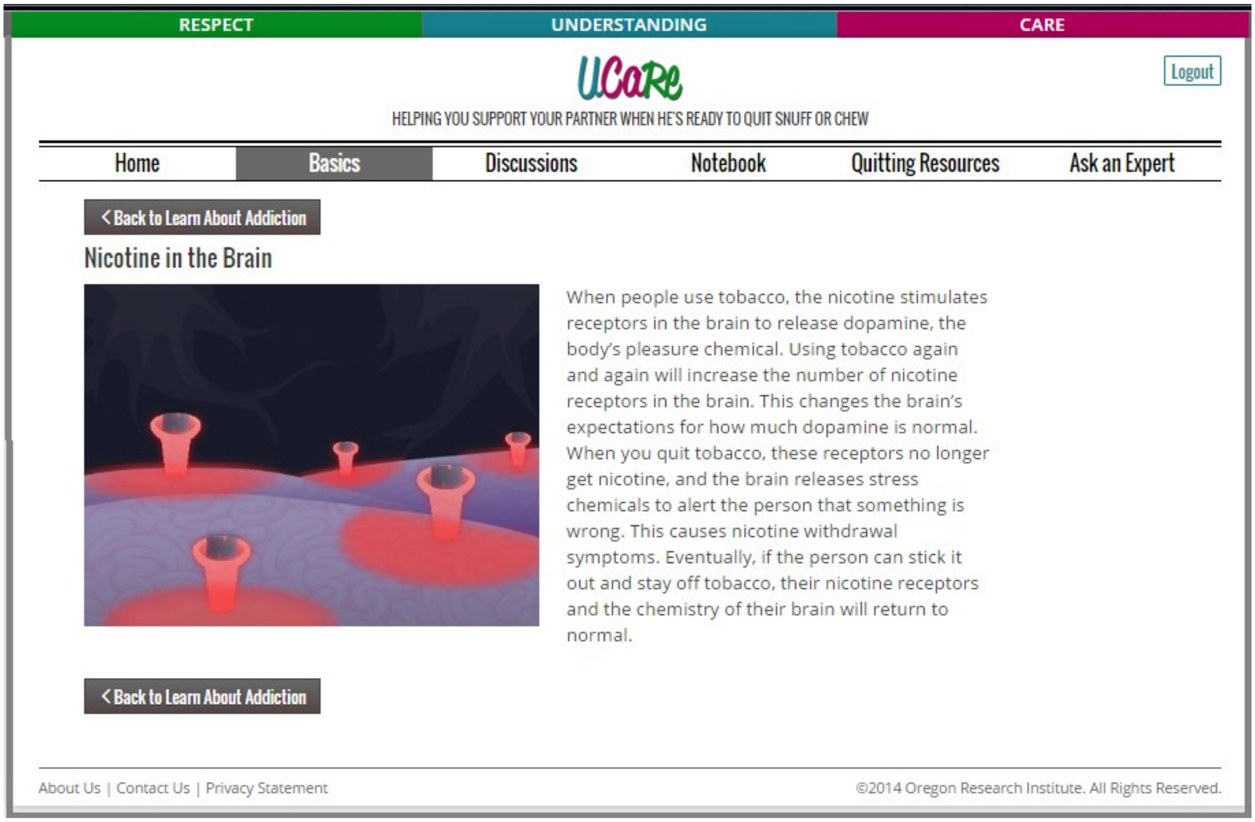

Figure A-3. “Megan’s Morning” Story

Users have the option to read the story, read while listening, or listen only.

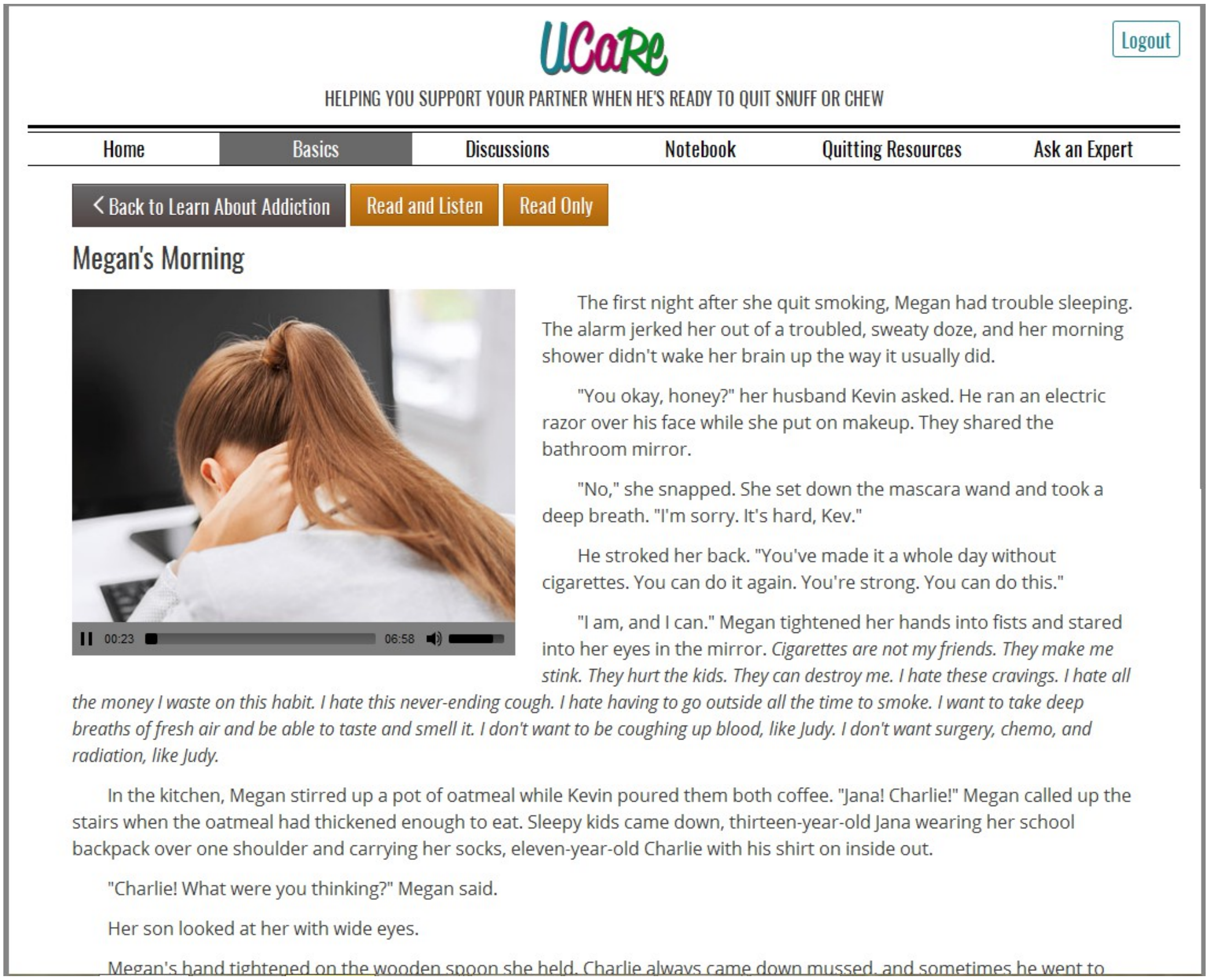

Figure A-4. The CONCENTRATE Game: Normal and Flashing Modes

During the normal mode, the user clicks on letters to spell out the word “concentrate.”

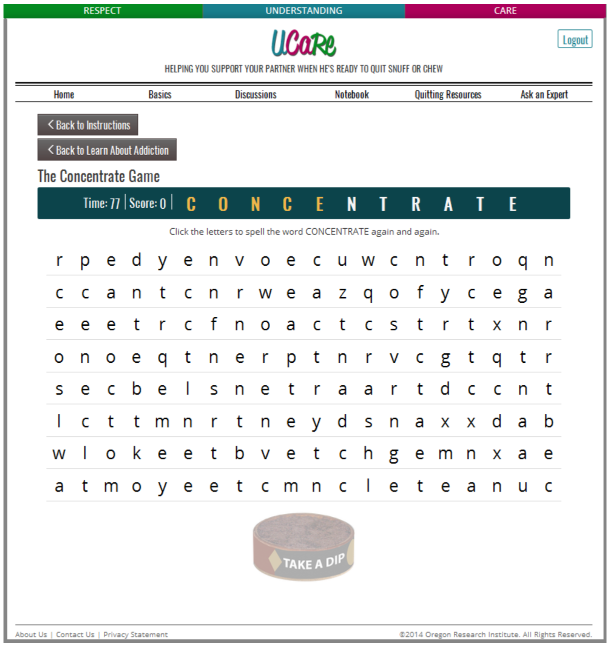

At regular intervals, the screen starts to flash distractingly, and the letters turn colorful and pulse in and out of view, making it much more challenging to find them. If the user clicks on the flashing “Take a Dip” can, the flashing goes away instantly and the normal view is restored. If the user is patient, the normal review is eventually restored without “taking a dip” as well.

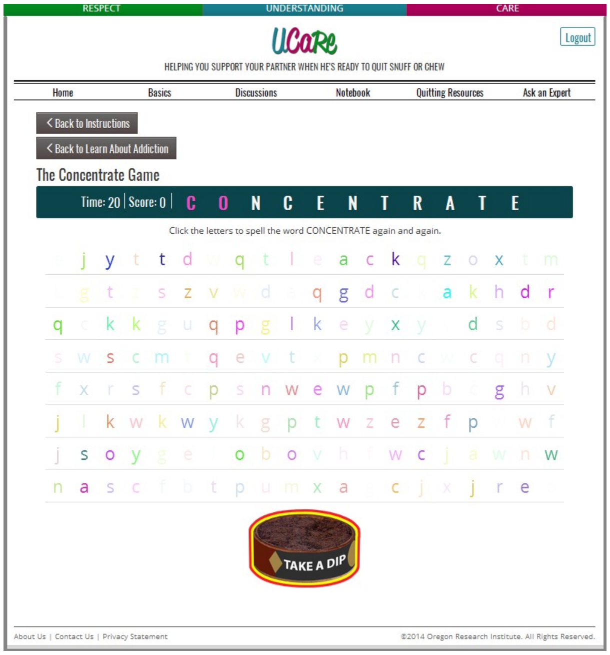

Figure A-5: My Notebook – Things to Do

My Notebook has two printable pages, Things to Remember (populated from the Take Notes box on Basics pages), and Things to Do (populated from the Basics checklist pages).

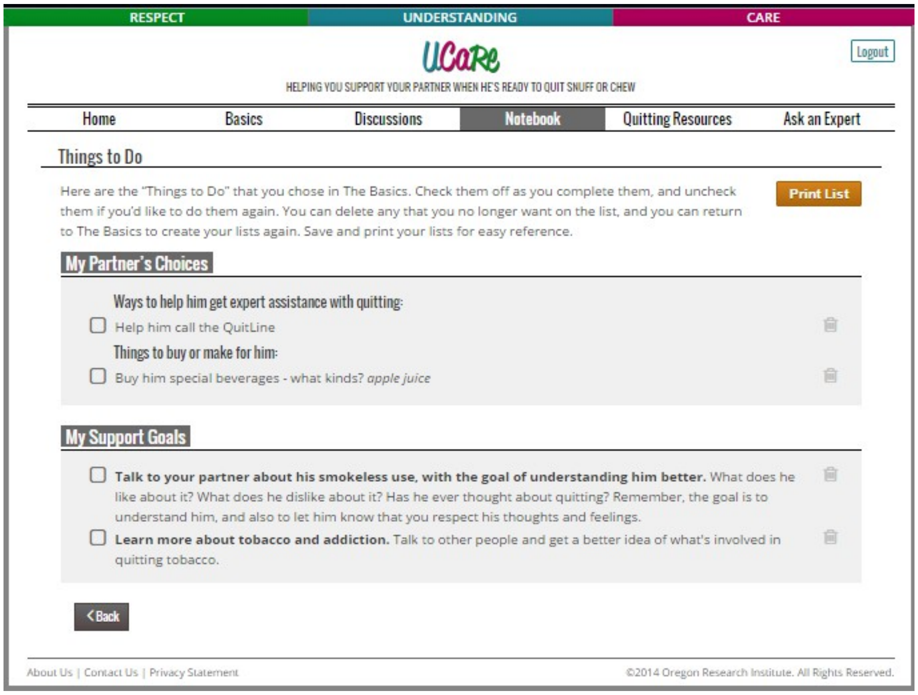

Figure A-6. UCare Basics: Sample Checklist Page

Choices made on checklist pages populated the “Things to Do” page in “My Notebook” (Figure A-6).

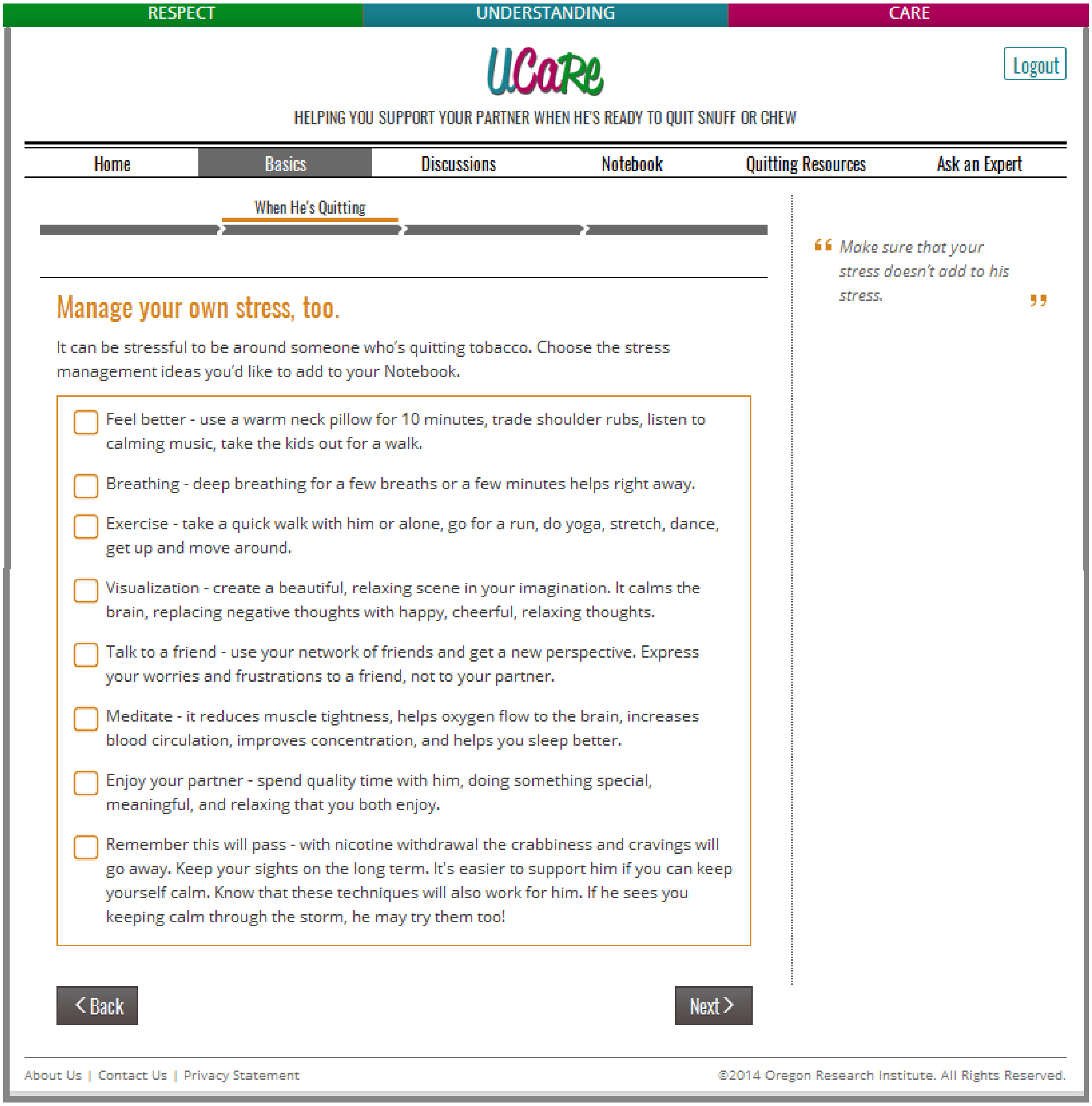

Figure A-7. Sample UCare Discussion Topic Page

The Discussion Topic pages address topics of special interest to some but not all users. Users have the option of commenting and creating a social community.

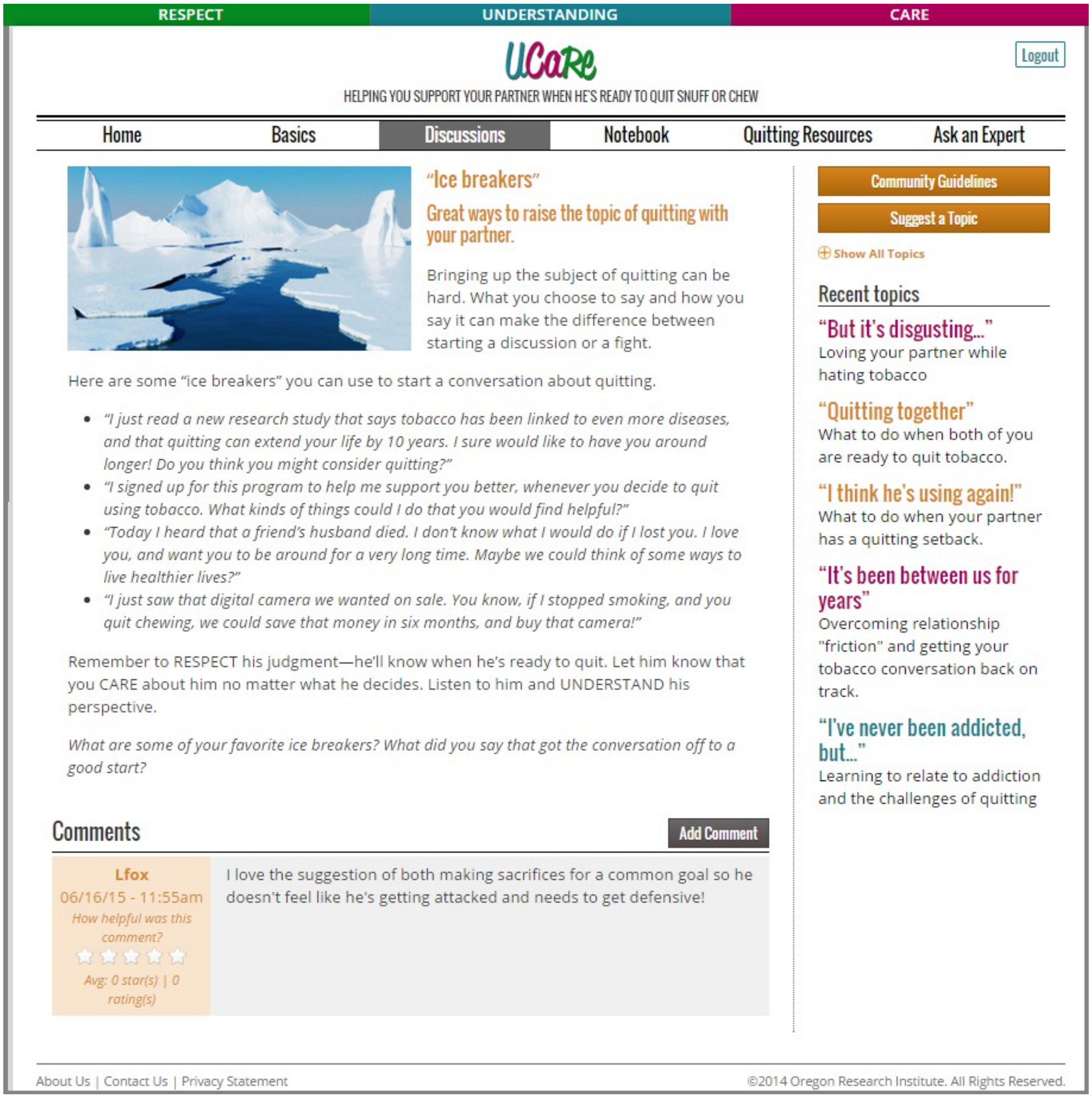

Supplement: Multimedia Appendix 1 [file formative_v2i1e10_app1.pdf]
